# Vaccine-induced responses to R21/Matrix-M – an analysis of samples from a phase 1b age de-escalation, dose-escalation trial

**DOI:** 10.3389/fimmu.2025.1620366

**Published:** 2025-06-26

**Authors:** Caroline Bundi, Duncan Bellamy, Elizabeth Kibwana, Lydia Nyamako, Rodney Ogwang, Kelvias Keter, Domtila Kimani, Ahmed M. Salman, Samuel Provstgaard-Morys, Lisa Stockdale, Adrian V. S. Hill, Philip Bejon, Ally Olotu, Mainga Hamaluba, Katie J. Ewer, Melissa C. Kapulu

**Affiliations:** ^1^ Centre for Geographic Medicine Research, Coast, Kenya Medical Research Institute-Wellcome Trust Research Programme, Kilifi, Kenya; ^2^ Ifakara Health Institute, Bagamoyo Research and Training Centre, Bagamoyo, Tanzania; ^3^ The Jenner Institute, University of Oxford, Oxford, United Kingdom; ^4^ Centre for Tropical Medicine and Global Health, Nuffield Department of Medicine, University Oxford, Oxford, United Kingdom

**Keywords:** malaria, vaccines, R21, immunogenicity, antibody, past exposure

## Abstract

**Introduction:**

The pre-erythrocytic malaria vaccine R21 vaccine adjuvanted with Matrix-M reported good efficacy (75%) in an ongoing phase 3 trial and was recommended World Health Organization for use in children 5–36 months. Vaccine-induced antibodies against NANP are associated with protection, however, various factors such as age, pre-existing immunity, and vaccine dose have been shown to influence vaccine responses.

**Methods:**

Samples from adults (n =18), children (n = 17), and infants (n = 51) vaccinated with R21/Matrix-M in a phase I trial were assayed for vaccine-specific antibody responses. We measured antibodies (quantity) by MSD and ELISA; and function (quality) by complement (C1q) fixation assay, inhibition of sporozoite invasion (ISI) assay, and avidity assay. Pre-existing malaria antibody exposure was assessed using an anti-3D7 *Plasmodium falciparum* crude parasite lysate ELISA.

**Results:**

Vaccine-induced CSP antibodies (against full-length R21, NANP, and C terminus), exhibited complement fixation and inhibition of sporozoites. These were significantly lower in adults compared to children and infants. Additionally, children had a higher rate of decay of vaccine-induced antibodies compared to adults 2 years post-vaccination. Furthermore, a higher Matrix-M adjuvant dose resulted in significantly higher C1q fixation, and ISI than the low adjuvant dose in infants. Importantly, functional measures ISI and C1q-fixation were positively associated with the vaccine-induced antibodies overall, but avidity was not. Interestingly, in adults, previous malaria exposure was negatively associated with ISI but positively correlated with avidity and C1q fixation. At baseline, all the study participants were seropositive for anti-HBsAg IgG above the WHO-required protective threshold of 10 mIU/mL, and titers significantly increased post-vaccination.

**Discussion:**

R21/Matrix-M was immunogenic across all age groups, with age and vaccine dose significantly affecting antibody magnitude and function. These findings emphasize the importance of evaluating the right adjuvant and vaccine dose for clinical development progression. This could thus inform the development of next-generation malaria vaccines. However, additional crucial factors need further exploration.

## Introduction

Malaria remains a major health issue, particularly for children under 5 in sub-Saharan Africa. In 2023, there were an estimated 246 million cases and 569,000 deaths in the WHO African Region (1). *Plasmodium falciparum*, responsible for 95% of global cases, is the deadliest species (2). While global mortality has decreased since 2000 due to measures like the use of insecticides and artemisinin drugs (1) resistance to these treatments (3), along with vector behavioral (4) and climatic changes (5, 6) threatens progress. Malaria control programs have also strained the global economy (7). Thus, adding effective malaria vaccines to existing strategies is a key global health priority.

Several malaria vaccines targeting different parasite stages (pre-erythrocytic, blood, and mosquito) are in development (8, 9). Notably, the WHO recommended two vaccines for use in regions of moderate to high malaria endemicity. The first, RTS, S/AS01E (Mosquirix), was pre-qualified by the WHO in 2021 (10). In Phase 3 clinical trials, where SMC was administered and age-based vaccination was used, it demonstrated a moderate efficacy of 59% against clinical malaria in children aged 5–17 months (11). Despite not reaching the WHO’s 75% efficacy goal, it has contributed to significant malaria control (12). The second, R21/Matrix-M, was pre-qualified in 2023 (13), with a remarkable 77% efficacy in Phase 2 clinical trial with a four-dose regimen for children aged 5–17 months when given in combination with SMC and with vaccination timed to coincide with the start of the malaria transmission season to maximize efficacy (14). It also showed high efficacy in areas with seasonal (75%) and perennial malaria transmission (68%) (15). However, the ongoing deployment of malaria vaccines is currently limited to age-based deployment, and these point estimates of efficacy using seasonal administration have limited relevance to real-world impact. Both vaccines face challenges with waning immunity over time, highlighting the need for developing strategies to sustain protection (15, 16).

Globally, it is estimated that of the 4–19 million children born each year (3-13% of the birth cohort), receive routine childhood vaccinations against infections such as measles, tetanus, and diphtheria, but remain inadequately protected against the target disease as a result of either limited coverage or vaccine effectiveness (17, 18). Pre-existing immunity is another factor that has been shown to influence vaccine response. Infants vaccinated with the rotavirus vaccine who had high titers of maternal anti-rotavirus IgG had lower seroconversion after vaccination (19, 20). This was also observed in RTS,S phase 3 study where children with high levels of anti-CSP antibodies at baseline had lower vaccine-induced antibodies post-vaccination (21). Previous studies in malaria-endemic regions have shown that malaria exposure could impair the immune system (22–24) which in turn may affect vaccine response (16). However, currently, the impact of previous malaria exposure on R21 vaccine responses is unknown.

Immune responses to malaria vaccines vary by age. In RTS,S infants aged 6–12 weeks had lower vaccine efficacy compared to older children 5–17 months (12). However, lower responses have been reported in adults compared to children in malaria-exposed volunteers post-vaccination with RTS,S (25, 26), METRAP (27, 28), and R21 (29) vaccines. Vaccination dose (both the protein and the adjuvant) and schedule have been shown to impact RH5 (30–32), yellow fever (33), R21 (34), and SARS-CoV-2 vaccine responses (35). In R21 vaccinated UK adults, there was no significant difference between the 10 µg and 50 µg vaccinated group at the peak time point (Day 84). However, at a late point (~6 months), the 50µg group had lower NANP antibodies compared to the 10µg group (36). In Burkina Faso, infants vaccinated with the R21 vaccine, the low adjuvant group (25 µg Matrix-M) had lower anti-NANP IgG responses compared to the high adjuvant group (50µg) (34). Previously, it was reported that there was no significant difference in the NANP antibodies between the different vaccination doses (adjuvant/protein) in infants’ groups in Kenya (Phase 1b) (29). However, this study had a smaller sample size (n~15) compared to Phase 2b in Burkinabe (n~150) (29, 34).

In the current study, we further investigated if there was a difference in the quality and/or magnitude of the antibodies against full-length R21 and C terminus that were not reported in the first study (29). The mechanism associated with R21 and RTS,S vaccine efficacy is not completely understood. Additionally, currently, there is no accepted correlate of protection for malaria vaccine candidates. In both R21 and RTS,S vaccines, the magnitude of anti-NANP IgG has been associated with protection and was considered in WHO approvals (12, 15). However, in RTS,S other antibody features such as avidity, affinity, complement-fixing capacity, isotypes, subclasses and, binding to Fcγ receptors and cellular responses have been associated with protection (37–39). However, these factors remain unexplored in the R21 vaccine.

In this study, we sought to determine the effects of age, vaccination dose, and previous malaria exposure on R21/Matrix-M-induced immune responses among healthy Kenyan volunteers. Here we had the unique opportunity to study both the impact of age (the same vaccine and adjuvant dose were given to adults, children, and infants), as well as the impact of either vaccine and adjuvant dose in infants

## Methodology

### Study design

This study utilizes data and plasma samples from a Phase 1b, open-label, age de-escalation, dose-escalation trial conducted in Kilifi, Kenya (VAC073) (Clinicaltrials.gov (NCT03580824). The study recruited healthy adults (18–45 years), children (1–5 years), and infants (5–11 months) from a region of moderate malaria endemicity: Junju (40). The region experiences two high malaria transmissions seasons, May to August and October to December, during which parasite prevalence rises beyond 70%. However, during the dry season the transmission is stable with a parasite prevalence of 30% (40, 41). The study volunteers were randomized to receive a full or half dose of R21 and adjuvant Matrix-M vaccine at 0, 1, and 2 months and a booster dose at 1 year after the initial dose. However, due to the COVID-19 pandemic in 2020, adults and children were given the booster dose 2 years after the primary series. The study was conducted between 28th April 2019 and 14th June 2022 (29). The vaccines were administered by the intramuscular route to the left deltoid. The vaccination schedule and vaccine dosing are shown in **Table 1**.

**Table 1:** R21/Matrix M vaccination schedule, dose, and timepoints in Kilifi, Kenya.

Group (Age)	Volunteers (N)	Month 0	Month 1	Month 2	Booster (1/2 years)
Group 1A/B Adults (18–45 years)	18	10μg R21/50μg MM	10μg R21/50μg MM	10μg R21/50μg MM	10μg R21/50μg MM
Group 2A Young children (1–5 years)	3	5μg R21/25μg MM	5μg R21/25μg MM	5μg R21/25μg MM	5μg R21/25μg MM
Group 2B Young children (1–5 years)	17	10μg R21/50μg MM	10μg R21/50μg MM	10μg R21/50μg MM	10μg R21/50μg MM
Group 3A/C Infants (5–11 months)	18	5μg R21/25μg MM	5μg R21/25μg MM	5μg R21/25μg MM	5μg R21/25μg MM
Group 3B/D Infants (5–11 months)	18	10μg R21/50μg MM	10μg R21/50μg MM	10μg R21/50μg MM	10μg R21/50μg MM
Group 3E Infants (5–11 months)	15	5μg R21/50μg MM	5μg R21/50μg MM	5μg R21/50μg MM	5μg R21/50μg MM

The clinical trial was conducted according to the principles of the Declaration of Helsinki and Good Clinical Practice guidelines. Written informed consent for participation in this study was provided by adult volunteers whilst the children and infant participants’ parents or legal guardians provided consent for participation in this study, which allowed the use of the sample and data for the primary trial objective and future exploratory analysis. Clinical trial details are published elsewhere (29).

### Assays

#### Antigens used in the study

The Serum Institute of India donated the Full-length R21 protein. Jenner Institute, University of Oxford donated the C-terminus peptide, and NANP peptide. Full-length R21 protein was produced at the Serum Institute of India, while the C terminus and the NANP (consisting of 6 repeats (Asn-Ala-Asn-Pro) x 6)) peptides were commercially obtained by Oxford from ProImmune, Oxford, UK. The schizont extract was produced in-house at KEMRI as previously described (42, 43). Samples assayed in each assay are shown in **Supplementary Table 1**.

### Total IgG measured by MSD

The Meso-Scale Discovery (MSD) multiplex assay was developed for measuring the concentrations of multiple protein targets within a single, small-volume sample. The assay plates were pre-coated with 4 different antigens (NANP, C term, HBsAg, and full-length R21) arranged in independent spots on the base of each well. The assay was validated at the Jenner Institute (44).

The assay was conducted as described by Stockdale et al. (44). Briefly, all reagents were brought to room temperature (RT) before plates were blocked with casein, sealed and incubated with shaking for 30 mins at RT. After blocking, plates were washed with MSD wash buffer and 50 μL/well of reference standard, controls, and diluted samples were added to the plate. After washing, 50 μL/well of Detection SULFO-TAG Anti-human IgG antibody was added to the wells. The sealed plates were then incubated with shaking at RT for 1 hour. After washing, 150 μL/well of MSD GOLD Read Buffer B was added to each well and read using the MSD plate reader within 15 minutes. Antibody concentrations are calculated and reported using MSD Methodical Mind Software.

### Total IgG measured by standardized ELISA

This assay was conducted as previously described by (29). In brief, 96-well NUNC Immuno plates (Fisher) were coated overnight at 4°C with 50μLof R21 (1µg/ml), NANP6 (0.2 µg/ml), C-terminus (1.5 µg/ml) or schizont extract (1:500 dilution) carbonate bicarbonate coating buffer (Sigma). After incubation, plates were washed with washing buffer (PBS containing 0.05% TWEEN 20 ((Sigma)) and blocked with blocked with 100μLof Blocker Casein in PBS (Thermo Fisher Scientific) for an hour at room temperature (RT). After removing the blocking buffer, standard curve and internal controls were created in casein using a pool of high-titer volunteer plasma, and 50μLof each dilution was added to the plate in duplicate. Test samples were diluted in casein starting at a minimum dilution of 1:100, and 50μLwas added in triplicate. Plates were incubated for 2 hours at RT and washed in the washing buffer. Plates were incubated with 50μLof goat anti-human IgG (γ-chain) conjugated to alkaline phosphatase ((AP) (Sigma)) diluted at 1:1000 for 1 h at RT. After the final wash, the plates developed with 50μl of p-nitrophenyl phosphate (pNPP (Sigma)) at 1 mg/mL in diethanolamine buffer (DEA), (Pierce)). Optical density at 405 nm was measured using an ELx800 absorbance reader (BioTek) until the internal control reached OD_405_ of 1. The reciprocal of the internal control dilution giving an OD_405_ of 1 was used to assign an AU value of the standard. Gen5 ELISA software v3.04 (BioTek) was used to convert the OD_405_ of test samples into AU values by interpolating from the linear range of the standard curve fitted to a 4-parameter logistics model. This is after the software calculated the average of the triplicate well and subtracted the background (casein only well). Any samples with an OD_405_ below the linear range of the standard curve at the minimum dilution tested were assigned to a minimum AU value of 1.

### NANP complement-fixing (C1q) ELISA

The C1q assay detects the capacity of the antibody to initiate the complement cascade. A 96 well flat bottom Maxisorp (NUNC, Thermo Scientific 442404) was coated with 0.2µg/mL of six repeats of the amino acid NANP repeat ((Asn-Ala-Asn-Pro) x 6) with a cysteine residue added to the C-terminus (NANP6C) antigen overnight at 4˚C. The plates were then washed with PBS tween and blocked with 200μl/well of casein for 2 hours at 37˚C. The plate was washed 3 times in PBS tween and the test serum diluted 1:1000 in casein was added 50μl/well and incubated for 2 hours at room temperature. After incubation, the plate was washed 3 times in PBS tween and complement (Purified human C1q (Millipore 204876)) added 30μl/well for 30 minutes at RT. The plate was then washed, and α-Complement Abs (Rabbit α-C1q Abs (Beeson lab in-house)) was added (50μl/well) and incubated for 1hr at RT. The plate was then washed and incubated with HRP conjugated Abs (Goat α-Rabbit IgG HRP (Bio-Rad #1706515)) (50μl/well) for 1hr at RT. After incubation, the plate was washed and incubated substrate (TMB (Thermo Scientific, Invitrogen 002023)) (50μl/well) for 7 minutes at RT in the dark. Stopping solution (Sulphuric acid1M) 50μl/well. The ODs of the plates were read at 450nm on Bio-tek ELx800 Microplate Reader with Gen5 software. The final OD per sample was calculated by subtracting the background (casein-only wells) from the average of the duplicate wells of the test samples.

### Inhibition of sporozoite invasion assay

The ISI procedure was adapted from a previously described methods (45, 46). The modification included an increase of the sporozoite infection concentration from 10000 spz per well to 15000 spz per well, adding trypsin to human hepatocytic cell line (Huh7) to help remove the cells on the day of acquisition, and changing of culture media from R10 (made up of RPMI,10% Fetal Bovine Serum, pen/strep and glutamine) to D10 (made up of Dulbecco’s Phosphate Buffered Saline,10% Fetal Bovine serum, pen/Strep, and glutamine).

The ISI assay was performed over 3 days. A Huh7 cell line that was cultured for about two weeks was used. On the first day, cultured Huh7 were counted, and the concentration was adjusted to 30,000 cells in 100uL/well and added to a 96-well flat-bottomed sterile tissue culture plate. This was incubated overnight at 37°C, 5% CO^2^ incubator. On the second day, viable *GFP-labelled P. berghei (Pb)* sporozoites expressing *Pf* CSP at the *P. berghei* CSP locus (*P. berghei PfCSP@CSP*) were obtained by dissecting the salivary glands of infected *Anopheles stephensi* (As) mosquitos. The mosquitoes were provided by the Jenner Institute Insectary team. The dissection involved the removal of the three pairs of salivary glands that were pooled in a homogenizer in DMEM media and left on ice until ready to count. The salivary glands were homogenized and sporozoites were counted using a 40x magnification microscope and diluted to a concentration of 150,000 spz/ml in DMEM medium. On the third day, the plates containing cultured Huh7 cells were retrieved from the incubator. The culture medium from Huh7 cells was aspirated, serum/plasma diluted 1:50 in D10, and 100μl added into each well (final serum/plasma concentration 1% in D10 per well) along with 100μl of sporozoite dilution (15,000spz/ml). Samples assayed were baseline samples (before vaccination) and 1-month post the third vaccination (day 84). The samples were assayed in duplicate. Additionally, 6 negative controls (Cells only, Huh7 only wells) and 4 positive controls (Spz 1-4, containing Huh7 cells and sporozoites but no plasma/serum) were added. On the third day, after incubating for 20–26 hours at 37°C, 5% CO2 incubator, D10 media was aspirated and plates were washed twice with 100μl sterile dPBS. Thereafter, 50μl was added to each well and to increase trypsin activity on cells, the solution was gently mixed ~10–15 times using a manual multi-channel pipette. Trypsin was neutralized using ISI buffer 1 (dPBS with 10% FCS) after 10 minutes. The cells were then transferred to a 96-well V-bottom plate and centrifuged at 1800rpm (764g) for 5 minutes. Finally, they were resuspended in 80μl of ISI buffer 2 (dPBS with 1% FCS) and immediately acquired using a BD LSRII and FACSDiva v6.2. Just before acquisition, DAPI stain (1:1000 dilution) was added to each sample to stain dead cells. Data was analyzed in FlowJo software v10.9 (TreeStar Inc., Ashland, Oregon) using the gating strategy in (**Supplementary Figure 1**). The percentage of sporozoite inhibition was calculated for each sample (average of duplicate wells) based on the reduction in the percentage of infected cells compared with the infectivity controls (average of 4–6 wells).

### Anti-CSP IgG antibody avidity ELISAs

This assay was used to quantify antibody binding strength to an antigen. IgG antibody avidity against NANP, C term peptides, and full-length R21 recombinant protein was assessed by sodium thiocyanate (NaSCN)-displacement ELISA. As previously described (47) an ELISA plate was coated overnight as described above in the standardized ELISA. Test serum was diluted in casein buffer to an OD of 1 based on standard ELISA results (total IgG EU results initially measured for each sample) and 50μl was added in 16 wells of 96 well ELISA plate after blocking the plates. The plates were incubated for 2 hours at room temperature. Then 8M stock solution NaSCN diluted in PBS was added in increasing concentration down the plate (0M- 7M) and incubated for 15 minutes in the dark. Plates were washed and then followed by a secondary antibody (goat anti-human γ-chain whole IgG alkaline phosphatase conjugate). The plates were developed by adding p-nitrophenylphosphate at 1mg/mL in diethanolamine buffer. The plates OD at 405nm were read on Bio-tek ELx800 Microplate Reader with Gen5 software. The plates were allowed to develop until the OD of the wells of the top row of the plate (where no NaSCN was added) reached 1 (0.8-1.2) (time dependent on the antigen). The concentration of NaSCN required to reduce the OD_405_ to 50% of the top row (where no NaSCN was added) was used as a measure of avidity index (IC_50_). This was calculated as an average of the duplicate wells.

### Statistical analysis

Data were analyzed using GraphPad Prism version 10.1 for Windows (GraphPad Software Inc., California, USA). Kruskal-Wallis test with Dunn’s correction for multiple comparisons was used to compare mean antibody levels between more than two time points or groups, and determine the significance between the timepoints or groups. Comparisons between two time points or groups were conducted with Mann-Whitney tests. Spearman correlation was used to test correlation analysis. All p values (or adjusted p values for tests with Dunn’s correction) are two-tailed and were considered significant at the value of p < 0.05.

## Results

### Participant characteristics

The Phase 1b study in Kenya included 91 study participants who were randomized to receive different vaccine/adjuvant doses, as shown in **Table 1**. Three children who received 5μg R21/25μg Matrix-M were excluded from this analysis due to the small number and subsequent lack of statistical power to compare with the other groups. The adults’ mean age was 337 months (~28 years), the children were 34 months (~3 years), and the infants were 7 months (**Supplementary Table 2**). Since the study cohort was recruited from a region of moderate malaria endemicity, we measured anti-schizont Ig antibodies (a marker for previous malaria exposure) at baseline. As expected, the adults had higher exposure, and this was significant when compared with infants (**Supplementary Table 2, Supplementary Figure 2**). Additional details on the study volunteers are published elsewhere (29).

### R21/Matrix-M vaccine-induced robust anti-full-length R21, anti-NANP, anti-C terminus, and anti-HBsAg IgG in malaria-exposed volunteers

**Figure 1:**
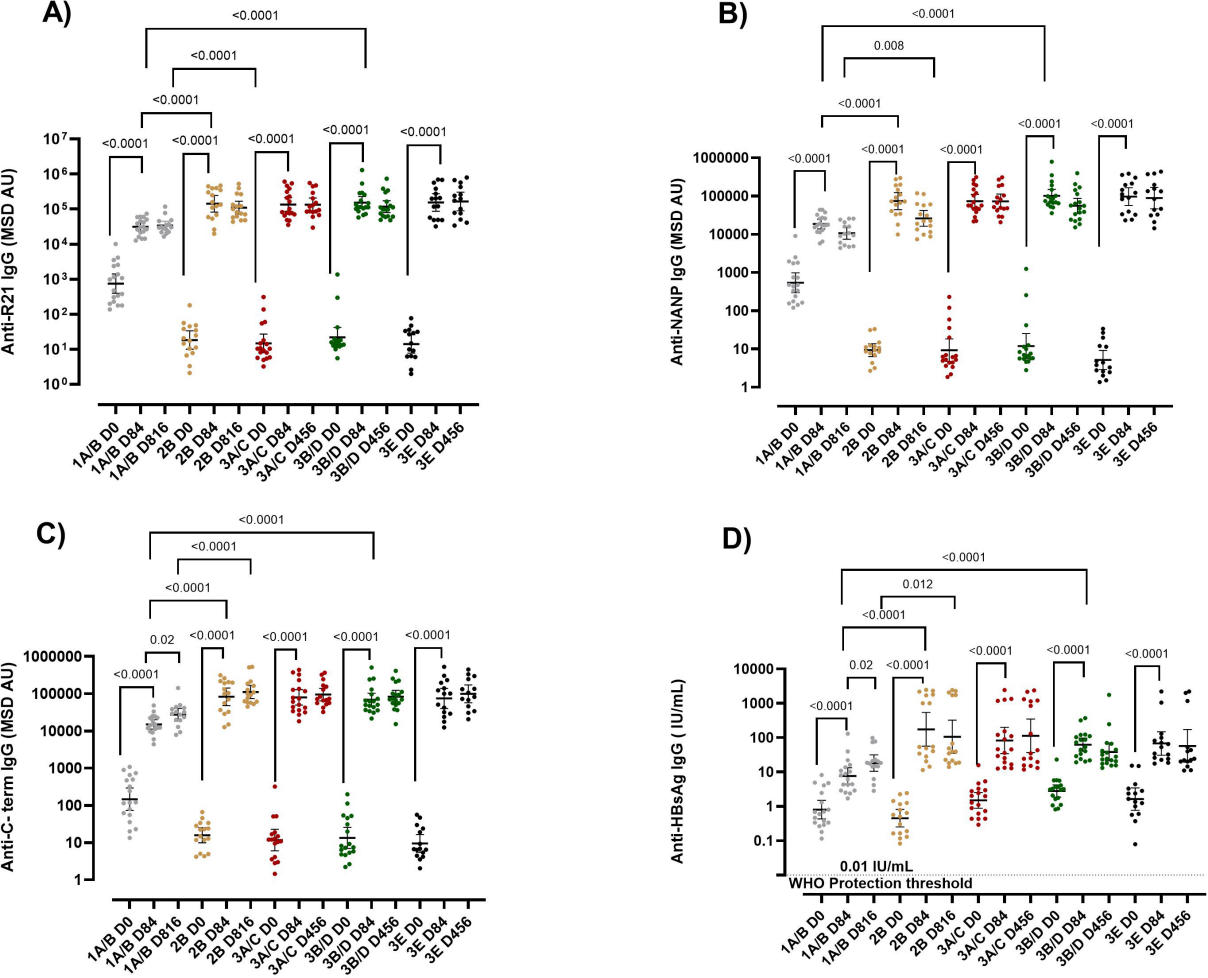
IgG responses post-vaccination with R21/Matrix-M. R21/Matrix-M vaccine-induced antibodies **(A)** Anti-full-length R21, **(B)** anti-NANP, **(C)** anti-C-term and **(D)** anti-HBsAg total IgG geometric mean (± 95% CI) as measured by MSD. The Y axis shows the geometric mean (± 95% CI) of antibody titers specified as log10 MSD antibody unit (AU). Kruskal-Wallis test with Dunn’s correction for multiple comparisons was used to compare mean antibody levels between more than two time points or groups, and determine the significance between the timepoints or groups. Sera were tested in triplicate. Comparisons between two time points or groups were conducted with Mann-Whitney tests. Only significance is indicated where p<0.05. The horizontal dotted line in panel **(D)** shows the 0.01 IU/mL WHO protective threshold for HBsAG. The x-axis shows the time course, D84 represents one-month post-primary vaccination, and D816 and D456 represent one-month post-booster dose for adults and infants respectively. The booster dose was administered at 1 year for infants and at 2 years for adults and children. Group 1A/B adults (10μg R21/50 μg Matrix-M), 2B children (10μg R21/50 μg Matrix-M), 3A/C infant (5μg R21/25 μg Matrix-M), 3B/D infants (10μg R21/50 μg Matrix-M), and 3E infants (5μg R21/50 μg Matrix-M). NANP-NANP6, (Asn-Ala-Asn-Pro) x 6, IgG, immunoglobulin G; HBsAg, Hepatitis B surface antigen; MSD, Meso-Scale Discovery multiplex assay.

We observed that the R21/Matrix-M vaccine was immunogenic in adults, children, and infants, as shown by a significant increase in IgG antibodies to the four vaccine components (full-length R21, NANP, C terminus, and Hepatitis B surface antigen (HBsAg) day 0 vs day 84 p = <0.0001 for all antigens) (**Figure 1**). Peak responses were recorded one month (Day 84) after the primary vaccination with Geometric Mean Titres (GMTs) of anti-full-length R21: 31253, 141817, 135734, 153301, 154794, anti-NANP: 18544, 73747, 73690, 101251, 96860, anti-C-terminus:15003, 82864, 79485, 68620, 74953 and anti-HBsAg: 7.69, 174.8, 83.15, 62.55, 68.01 for groups 1A/B, 2B, 3A/C, 3B/D and 3E respectively (**Figure 1**). The vaccine-induced antibodies waned over time, but a booster dose administered at one year in infants and at 2 years in adults and children restored the antibody levels to those seen at the peak after the primary series (**Figure 1**, **Supplementary Figure 3**). The GMTs post booster were anti-full-length R21: 33758, 109897, 132939, 117227, 165935, anti-NANP: 10631, 26093,72976, 55610, 88431, anti-C-terminus:27303, 110832, 94869, 83440, 98872 and anti-HBsAg: 18.22, 106.2, 112.9, 38.37, 57.52 for groups 1A/B, 2B, 3A/C, 3B/D and 3E respectively. At baseline, all the study participants were seropositive for anti-HBsAg IgG (> 0.01IU/mL) (**Figure 1D**).

### R21/Matrix-M induced lower antibody levels in adults compared to infants, with vaccination dose having no influence on magnitude

When we compared the vaccine response by age, at baseline, adults had significantly higher anti-full length R21, anti-NANP, and anti-C-terminus antibodies compared to children and infants (**Figures 1A–C**). Interestingly, post-vaccination for those receiving the same vaccine dose, adults had substantially lower vaccine-induced antibodies than infants and children at all time points (adults (1A/B) vs infants (3B/D) and, adults (1A/B) vs children (2B) p = < 0.05 for all antigens) (**Figure 1**). Adults and children received a booster dose at 2 years of age, whereas infants received their booster at 1 year. Due to this difference in the timing of the booster, a direct comparison of post-booster immune responses between infants and the older age groups (children and adults) were not conducted.

We further compared the vaccine response in the three infant groups that received different doses of R21/Matrix-M (3A/C (5μg R21/25 μg Matrix-M), 3B/D (10μg R21/50 μg Matrix-M), and 3E (5μg R21/50 μg Matrix-M). No significant difference was observed in the magnitude of the vaccine-induced antibodies between the three infant groups (p = > 0.05 for all antigens at all time points) (**Figure 1**, **Supplementary Figure 3**). This indicated that the magnitude of the vaccine-induced antibodies was influenced by age but not by the vaccine dose in this cohort.

### The percentage durability of the R21/Matrix-M vaccine-induced antibodies was higher post-booster dose compared to post-primary vaccination

We calculated the percentage durability of the vaccine-induced antibodies after the primary and post-booster doses. The percentage durability after primary vaccination was calculated by dividing the antibodies pre-booster dose at 2 years (in children and adults group) or 1 year (in infant groups) by the peak antibodies (day 84), then multiplied by 100. The percentage durability after the booster dose was calculated by dividing antibodies 1-year post booster dose (i.e. 3^rd^ year for the children and adults, and 2^nd^ year for the infants) divided by antibodies at one-month post booster dose (Day 456 for infants or Day 816 for adults and children), multiplied by 100. Overall, it was surprising to find that in children and infants groups, the antibody percentage durability (full-length R21, NANP, and C-terminus) was significantly higher post the booster dose compared to post the primary vaccination, however, this was not significant in adults (1A/B) and group 3A/C for anti-NANP antibodies (**Supplementary Figure 4**). Indeed, at 2 years (infants) and 3 years (children and adults), the vaccine-induced antibodies remained significantly higher compared to baseline (**Supplementary Figure 3**). However, we also noted that a few volunteers had a percentage durability greater than 100% for anti-full length R21 and anti-NANP (**Supplementary Figures 4A, B**).

### The percentage durability of the R21/Matrix-M induced antibodies was higher 2 years post-primary vaccination in adults than in children

When comparing by age, for those receiving the same vaccine dose, the percentage durability rate of vaccine-induced anti-full length R21, anti-NANP, and anti-C-term antibodies was higher in adults (1A/B) compared to children (2B) after the primary vaccination. The median percentage durability post-primary vaccination were anti-full length R21 17.3% vs 6.7% p = <0.0001, anti-NANP 38.3% vs 6.9% p = 0.006, anti-C terminus 24.8% vs 5.0% p = 0.0001 in adults (1A/B) versus children (2B), respectively (**Supplementary Figure 4**). The comparison couldn’t be made between adults and infants, or children and infants, due to differences in sampling and booster dose administration time points. Similarly, the three infant groups that received different doses of R21/Matrix-M (3A/C received 5μg R21/25μg Matrix-M, 3B/D received 10μg R21/50μg Matrix-M, and 3E received 5μg R21/50μg Matrix-M showed no significant difference in the percentage durability of the vaccine-induced antibodies post-primary vaccination or post-booster (**Supplementary Figure 4**).

### R21-induced IgM and IgA were significantly lower in adults compared to infants and children

We further evaluated anti-full length R21 and anti-NANP IgM and IgA measured by ELISA. There was no significant difference in the anti-full length R21 and anti-NANP IgA and IgM between the three infant groups (Day 84) (**Supplementary Figures 5A–D**). Like vaccine-induced IgG, anti-full-length R21 IgA and IgM for those receiving the same vaccine dose were significantly lower in adults than in children and infants. Similarly, anti-NANP IgA was significantly lower in adults compared to children and infants, but anti-NANP IgM was only significantly different between adults and children (**Supplementary Figures 5A–D**). This indicates that age, but not the vaccination dose, also influenced the magnitude of R21/Matrix-M vaccine-induced IgM and IgA antibodies.

### Lower R21/Matrix-M vaccination dose induced antibodies with lower complement fixing capacity than the higher dose

The quality of the vaccine-induced antibodies has been shown to play a vital role in vaccine-induced protection. Here, we evaluated the function of the R21/Matrix-M induced immunity using three functional assays: the Complement fixing assay (C1q) ELISA, the inhibition of sporozoite assay (ISI), and avidity ELISA.

**Figure 2:**
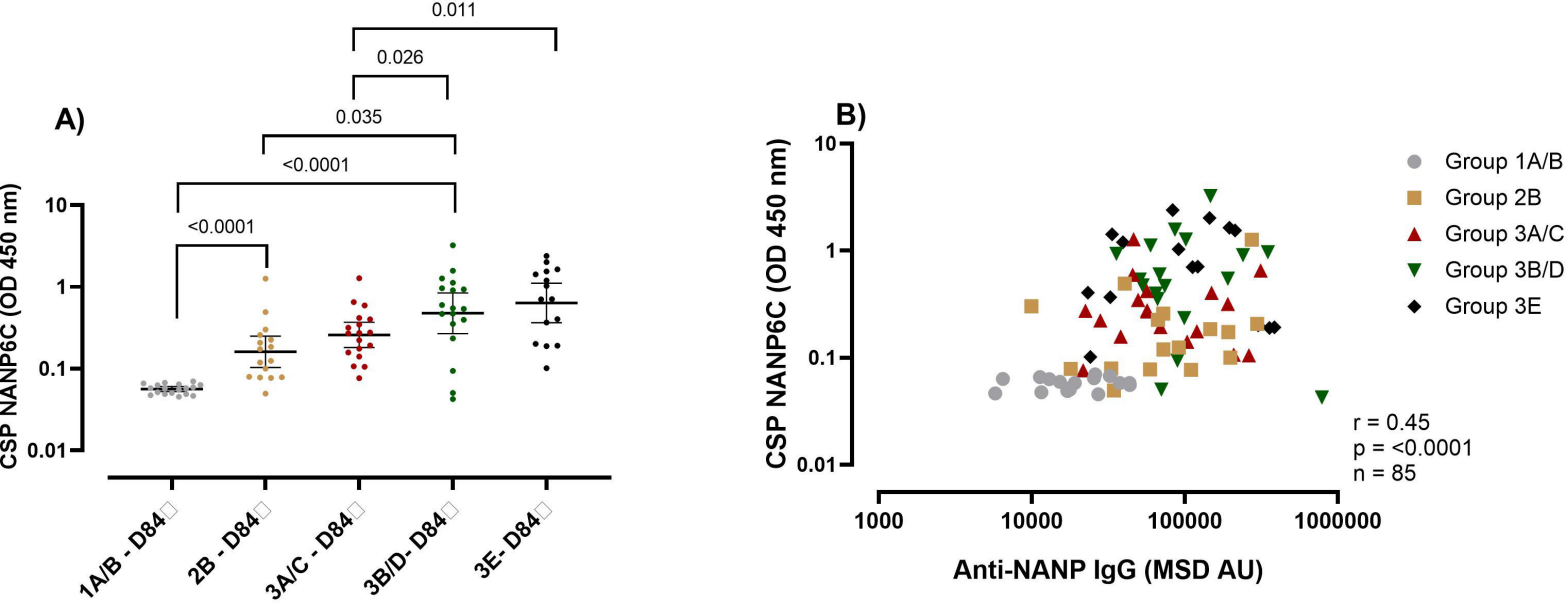
Complement fixing capacity of the R21/Matrix-M vaccine-induced NANP antibodies. **(A)** The plots show anti-NANP complement fixing capacity (C1q) OD geometric mean (± 95% CI) at day 84 (1-month post-primary vaccination). **(B)** Correlation between the NANP C1q and NANP magnitude at day 84 (1-month post-primary vaccination). Kruskal-Wallis test with Dunn’s correction for multiple comparisons was used to compare C1q between more than two time points or groups, and determine the significance between the timepoints or groups. Only significance is indicated where p<0.05. The correlation was computed with Spearman rank correlation. Group 1A/B-10μg R21/50μg Matrix M, group 2B- 10μg R21/50μg Matrix M, group 3A/C 5μg R21/25μg Matrix M, group 3B/D10μg R21/50μg Matrix M and group 3E 5μg R21/50μg Matrix M. OD, optical density; MSD, Meso-Scale Discovery multiplex assay, NANP- NANP6, (Asn-Ala-Asn-Pro) x 6.

Using groups receiving the same vaccine and adjuvant dose (10μg R21/50μg), we observed that the adults (Groups 1A/B) (Geometric Mean (GM) O.D 0.056 (95% CI 0.05-0.06)) had the lowest C1q binding capacity, followed by children (Group 2B) (GM O.D 0.16 (95% CI 0.10-0.24)) and infants (Groups 3B/D) (GM O.D 0.48 (95%CI 0.26-0.84) had the highest C1q binding capacity (**Figure 2A**) at one-month post-primary series of vaccinations (Day 84).

When we compared infants that received different vaccination dose, surprisingly we found that the infant group that received the low vaccination dose (Group 3A/C 5μg R21/25μg) had significantly lower C1q capacity (GM 0.26 (95%CI 0.18-0.37)) compared to those that received double the vaccine antigen or adjuvant respectively (Group 3B/D 10μg R21/50μg GM OD 0.48 (95%CI 0.26-0.84)) and (Group 3E 5μg R21/50μg GM OD 0.64 (95% CI 0.37-1.12)) p = 0.026 for 3A/C vs 3B/D and p = 0.011 for 3A/C vs 3E (**Figure 2A**). This was unexpected since we had observed no difference in the magnitude of the vaccine-induced antibodies between the infants’ groups (**Figures 1A–D**). Correlation showed an overall significant positive association between NANP total IgG antibodies and C1q complement fixing (r = 0.45, p = <0.0001) (**Figure 2B**).

### A lower R21/Matrix-M vaccination dose and adults were associated with lower sporozoite-inhibiting vaccine-induced immunity

The second functional assay we evaluated was the ISI. In adults, we measured the ISI at baseline for all the participants since they had significant levels of antibody responses at baseline (**Figures 1A–C**). R21/Matrix-M vaccination resulted in a reduction in sporozoite invasion in adults; we observed an average inhibition of 28.9% at baseline and 40% at day 84 compared to the positive controls, (p = 0.012) (**Supplementary Figure 6A**).

**Figure 3:**
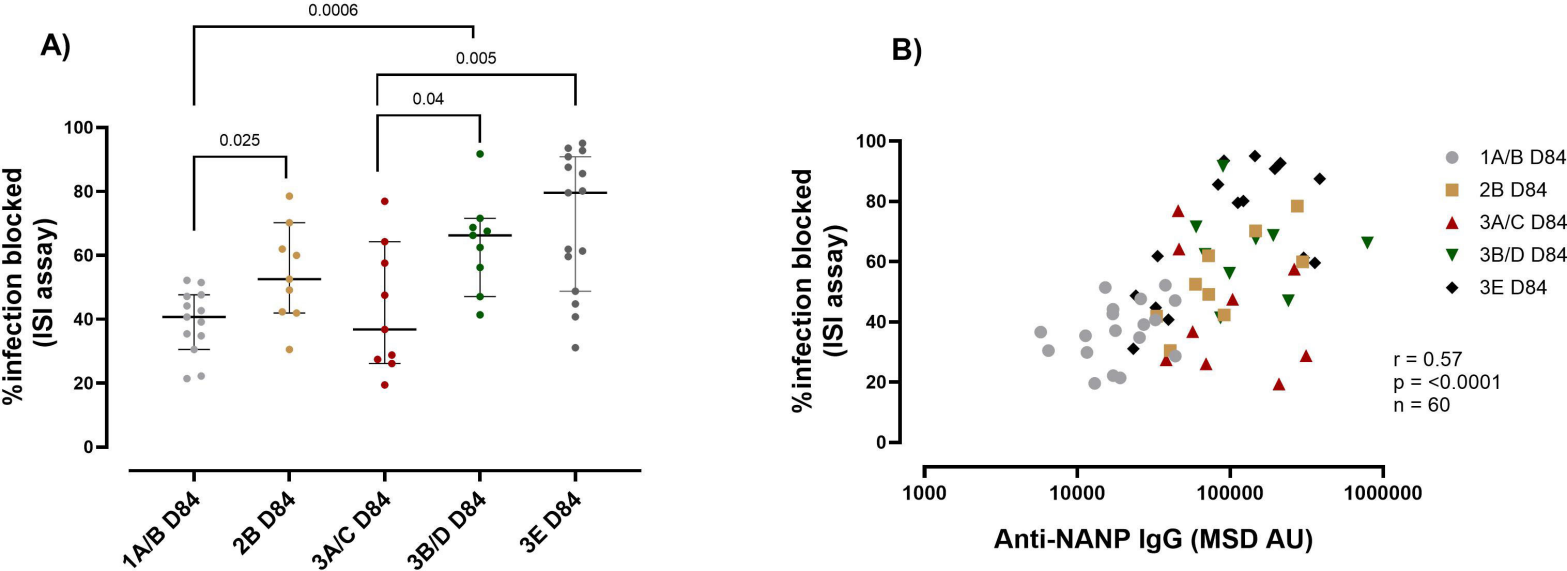
R21 induced immunity sporozoite inhibition capacity by age and vaccine dose post-vaccination. **(A)** The plots show the percentage of infection blocked as assayed by Inhibition of sporozoite (ISI) assay median (± 95% CI). **(B)** Correlation between the ISI and NANP magnitude at day 84 (1-month post-primary vaccination). Kruskal-Wallis test with Dunn’s correction for multiple comparisons was used to compare ISI between more than two time points or groups, and determine the significance between the timepoints or groups. Only significance is indicated where p<0.05. The correlation was computed with Spearman rank correlation. Group 1A/B-10μg R21/50μg Matrix M, group 2B- 10μg R21/50μg Matrix M, group 3A/C 5μg R21/25μg Matrix M, group 3B/D10μg R21/50μg Matrix M and group 3E 5μg R21/50μg Matrix M. OD, optical density, MSD, Meso-Scale Discovery multiplex assay, NANP-NANP6, (Asn-Ala-Asn-Pro) x 6.

Due to the low throughput for the ISI assay, not all pre-vaccination samples could be included. We observed a trend of increased reduction in sporozoite invasion post-vaccination compared to baseline for each group, where pre-vaccination samples were included (**Supplementary Figure 6A**). As was observed with respect to antibody magnitude, for those receiving the same vaccine dose adults (group 1A/B) had significantly lower (median 40.68%) sporozoite-inhibiting capacity than children (2B) (median 52.57%) and infants (3B/D) (Median 66.2%) (1A/B vs 2B p = 0.025, 1A/B vs 3B/D p = 0.0006) (**Figure 3A**). As with C1q, the dose influenced the capacity of the vaccine-induced immunity to inhibit sporozoite invasion. Infants (Group 3A/C 5μg R21/25μg) that received a lower Matrix M dose had significantly lower sporozoite inhibitory capability (median 36.8%) compared to the higher Matrix M group (Group 3E 5μg R21/50μg Matrix M) (median 79%) p = 0.005 (**Figure 3A**), indicating an improvement with increased adjuvant dose. Further analysis showed that the vaccine-induced anti-NANP significantly correlated with the ISI (anti-NANP *r* = 0.57, p = <0.0001) (**Figure 3B**).

### A booster dose increased the avidity of R21/Matrix-M-induced IgG antibodies

The third functional assay we evaluated is the avidity ELISA. This assay evaluates the total binding strength of the vaccine-induced antibodies to their target antigen. Here, we observed that there was a trend to an increase in avidity post-booster dose, and this was significant for anti-full length R21 and anti-C-terminus (**Supplementary Figures 7A–C**). Unexpectedly, within the same vaccine/adjuvant dose, children (2B) had the weakest antibody avidity compared with adults (1A/B) and infants (3B/D) for any of the three anti-CSP antibodies tested (**Supplementary Figures 7A–C**). Additionally, there was no difference in antibody avidity between the three infant groups that received different vaccine doses (**Supplementary Figures 7A–C**). We equally did not see an association between anti-full length R21, anti-NANP, and anti-C terminus IgG and their respective antibody avidities (**Supplementary Figures 7D–F**). In summary, we found that the vaccine dose influences the quality of the vaccine-induced immune response but not the magnitude. However, age influenced the magnitude and the quality of the R21-induced immunity.

### Effects of malaria exposure on R21 vaccine response

**Figure 4:**
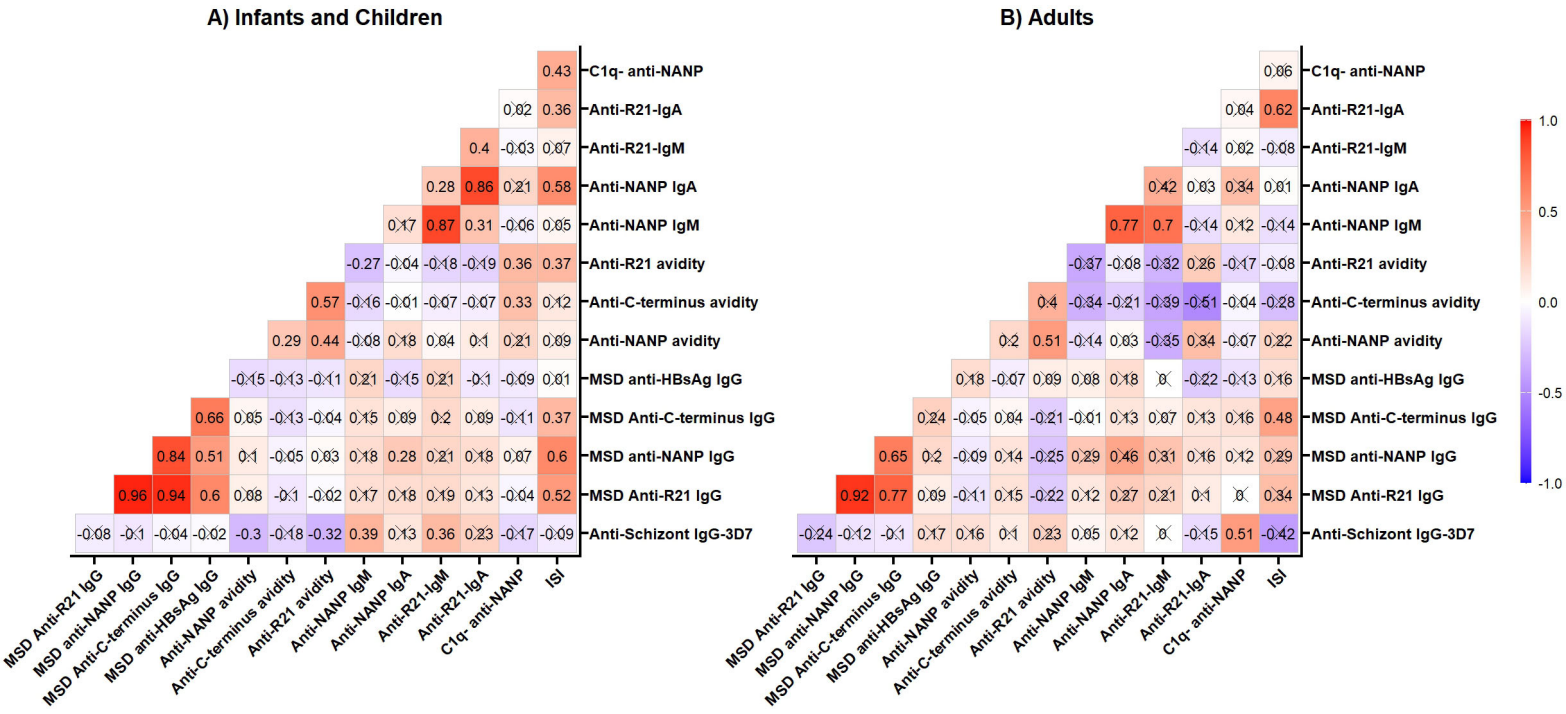
Correlation matrix. Anti-schizont IgG antibodies were measured at baseline by ELISA; all the other vaccine-induced response was measured on Day 84 (a month post-primary vaccination). **(A)** Infants -group 3A/C (5mg R21/25mg Matrix M), group 3B/D (10mg R21/50mg Matrix M) and group 3E (5mg R21/50mg Matrix M) and Children- Group 2B (10mg R21/50mg Matrix M); **(B)** Adults- Group 1A/B (10mg R21/50mg Matrix M). The cross (X) indicates correlations that are not significant at 0.05 alpha level. The correlation was computed with Spearman rank correlation. OD, optical density; MSD, Meso-Scale Discovery multiplex assay, NANP-NANP6, (Asn-Ala-Asn-Pro) × 6.

We measured anti-schizont Ig antibodies (a marker for previous malaria exposure) at baseline (**Supplementary Table 2**, **Supplementary Figure 2**). Here, we assessed the effects of the previous malaria exposure on the R21 vaccine responses using a correlation matrix. In infants and children, previous malaria exposure (anti-schizont IgG) had a weak correlation that was significant with anti-full-length R21 and anti-NANP avidity (**Figure 4A**). Surprisingly, anti-NANP and anti-R21 IgM were also significantly associated with previous malaria exposure in children and infants. In adults, previous malaria exposure was significantly associated with C1q (**Figure 4B**). When we conducted multivariate regression analysis adjusting for age, sex, and vaccination dose, we did not find any significant association between previous malaria exposure and R21 vaccine response immune markers (which could be due to small sample size) (data not shown).Discussion

It has previously been demonstrated that the R21 malaria vaccine generates high-quality antibody responses that provide protection to vaccinees in clinical trials. In this study, we focused on evaluating how age, vaccination dose, and naturally acquired pre-existing anti-malarial immunity impacted R21/Matrix-M humoral responses and function. We have shown that adults from a region of the malaria-endemic region have naturally acquired low levels of anti-R21, anti-NANP, and anti-C term antibodies. In addition, adults had lower responses of R21/Matrix-M: magnitude and function than children and infants. Surprisingly, we also observed that the vaccination adjuvant dose greatly impacted the function of the vaccine-induced antibodies among infants.

Vaccine-induced antibody levels and function (ISI and C1q) were lower in adults compared to the infant and child groups. This age-different response has also been reported previously for malaria and vaccines against other diseases. In R21/Matrix-M vaccine trials in West Africa, vaccine-induced antibodies were significantly lower in Burkinabe adults than in children (14, 48). Similarly, in ChAd63-MVA ME-TRAP, a pre-erythrocytic vaccine, 10-fold higher anti-TRAP antibody levels were reported in West African infants as compared to UK adults and West African adults (27). While direct comparative RTS,S studies between adults and children are limited, the available data provide some insights into age-related differences in efficacy and immunogenicity. An estimated efficacy of 47% in 15-week surveillance, was reported in malaria-exposed adults in Phase 1 (49), and an efficacy of 55.8% against clinical and severe malaria in a one-year follow-up was reported among children 5–17 months in Phase 3 (50). Although this needs to be interpreted with caution due to the differences in RTS,S trial setting. The cause of this difference is not clearly understood, speculation about the immune tolerance due to chronic malaria exposure has been reported, which could lead to vaccine hypo-responsiveness in adults (24, 51–54). Moreover, adults showed some level of ISI and antibodies at baseline, likely associated with previous malaria exposure, in line with high anti-schizont IgG antibodies observed in adults at baseline. Interestingly, children from malaria-endemic regions have reported higher malaria vaccine responses compared to malaria naïve adults (29, 32, 47, 48). This indicates that although immune tolerance due to malaria exposure could contribute to malaria vaccine hypo-responsiveness, more research is required to understand the effect of age on malaria vaccine response. Moreover, differences in vaccine response have been observed between infants and older children. For example, in RTS, S, infants (6–12 weeks) were found to have lower efficacy than older children (12, 25). These effects of age have been observed in other vaccines such as the COVID-19 vaccine (ChAdOx1-nCoV19 (AZD1222)) younger children (aged 6–11 years) were reported to induce higher antibodies than older children (aged 12–17 years) (55). This has been thought to be driven by immune system maturation, whereby older children have a more mature immune system compared to younger children. In the current study, we did not observe any difference in the magnitude of R21/Matrix-M antibodies between infants and children. However, the effects of age on vaccine response remain undetermined. More mechanistic studies are required to give a deeper insight into differential responses in vaccines between infants, children, and adults.

At baseline, most children and infants in the cohort lacked vaccine-induced antibodies, with the exception of anti-HBsAg IgG (> 0.01IU/mL), likely reflecting routine early-life hepatitis B vaccination (56) or prior exposure among adults (57). Although baseline responses were observed, a significant post-vaccination increase in anti-HBsAg IgG was detected across all participants. Due to the limited sample size, the influence of baseline antibody presence on vaccine responses was not assessed in this study. However, prior data from RTS,S trials indicate that baseline immunity can affect vaccine immunogenicity (58). We recommend that future studies with larger cohorts, such as Phase 3 trials, should incorporate this important analysis.

In this study, varying the vaccination dose (R21 antigen or Matrix-M adjuvant) in infants did not significantly impact the magnitude of the antibody response or durability, but had impact on (ISI and C1q). However, in West Africa, in larger studies a 25ug Matrix-M dose was associated with significantly lower vaccine-induced antibodies than a 50ug dose, using the same (5ug) dose of R21 (14, 34). It could be in the current study, we observed no significant difference in the antibody levels between the different doses due to the small sample size per group (~ n=15) as compared to the larger West Africa study (~n=150) (14, 34). Similar phenomena were observed in the R21/Matrix-M vaccine, where a fifth (10 µg vs 50 µg) of the vaccine-induced a similar vaccine as a full-dose response in UK adults (48). In the current study, the ISI and C1q activity was higher in the high Matrix-M (50ug) infants compared to the low Matrix-M (25ug) infants. This is in line with previous reports where Matrix-M has been indicated to enhance the quality of the antibody responses as well as induce cellular responses. This indicates the importance of an adjuvant dose in vaccine immunogenicity (59). Obtaining optimal vaccine doses is essential as reduced vaccine dose has dose-sparing and cost-saving implications for vaccine production, particularly if vaccine supply is limited.

One main challenge facing the malaria vaccine is maintaining the vaccine-induced immunity (12, 14, 16). In this study, antibodies waned post-vaccination; however a booster dose restored the response. Additionally, the C term and full-length R21 antibody avidity increased in infants and children post-booster dose but not NANP antibody avidity. This could be indicating antigen-specific differential response to boosting. Improved immune response with boosting has been reported in R21 vaccinated volunteers in West Africa, in RTS,S, and in the SARS-CoV-2 vaccine (14, 60, 61). The percentage durability of the vaccine-induced antibodies was lower in children compared to adults. Additionally, the percentage durability of the vaccine-induced antibodies was lower one-year post-primary vaccination compared to the one-year post-booster dose in infants. This could be indicating the benefit of a booster dose. We speculate that a booster dose induces more durable antibodies due to the improved germinal center activities and the existing memory B cells that lead to the production of high-quality antibodies that are more durable (62–64) In the RH5/Matrix-M vaccine, delaying the third dose by 6 months was associated with improved magnitude, function, and durability of the vaccine-induced immunity (32, 65). They similarly suggested that delaying the third dose could be associated with better GC activation and higher quality B cell response and plasma cells. Few infants and children had a percentage durability greater than 100% for anti-full-length R21 and anti-NANP. Although we do not have individual malaria exposure data for our participants, we hypothesize that, since they are living in a region of moderate malaria endemicity, they may be acquiring natural immunity that is boosting their responses.

Our study had a few limitations. First, we used data from the Phase 1b malaria vaccine trial, which involved small sample sizes (n=15–18 per group). While these numbers were adequate for assessing safety, they were limited for immunological analysis, which requires intergroup comparisons. Replication in larger cohorts and different settings is recommended. Second, we examined how age, malaria exposure, and dose influenced immune markers, but given the immune system’s complexity, future studies should consider a systems serology approach with larger sample sizes, such as in Phase 3 trials, to more comprehensively assess factors affecting the R21/Matrix-M vaccine response and identify potential correlates of protection.

The findings here underscore the importance of age, vaccine dose, and prior malaria exposure in modulating the immune response to the R21 vaccine. While both R21/Matrix-M and RTS,S are now WHO-prequalified for use in young children, significant knowledge gaps remain, particularly regarding optimal dosing strategies, vaccine scheduling, and efficacy in underrepresented groups such as infants under five months, pregnant women, and other at-risk populations. Future studies should prioritize evaluating modified vaccination regimens, including protein and adjuvant dose adjustments and delayed or fractional dosing schedules, to enhance efficacy, immunogenicity, and durability of protection. Fortunately, there is an ongoing study in Mali (ClinicalTrials.gov, NCT05155579), looking at immunization in 6-week-old infants and also evaluating non-interference with the existing childhood expanded program on immunization (EPI) schedule, whose results are eagerly anticipated. In addition, there is a planned study that aims to identify an optimal infant vaccine schedule for R21/Matrix-M malaria vaccine, which is better aligned with the timing of other vaccine interventions (ClinicalTrials.gov, NCT06879327). This will inform on how best to implement the vaccine in the real-world setting. Future R21 trials could focus on optimizing the vaccination schedule, vaccine, and adjuvant dose, as well as expand the target group to other groups at risk of malaria infection. However, the data from the current study, combined with Phase 2b were used to support the selection of the final dose used in the Phase 3 licensure study for R21 (15). The ongoing trials are expected to provide critical data to inform the design and deployment of second-generation malaria vaccines.

## Data Availability

The original contributions presented in the study are included in the article/**Supplementary Material**. Further inquiries can be directed to the corresponding authors.
