# Supplementary material for: Vaccine-induced responses to R21/Matrix-M – an analysis of samples from a phase 1b age de-escalation, dose-escalation trial
Source: Front Immunol. 2025 Jun 26;16:1620366. doi: 10.3389/fimmu.2025.1620366 (PMC12241110; doi:10.3389/fimmu.2025.1620366)
Supplement: Supplementary file 1 [file Table1.docx]

**Supplementary Figures and Tables**


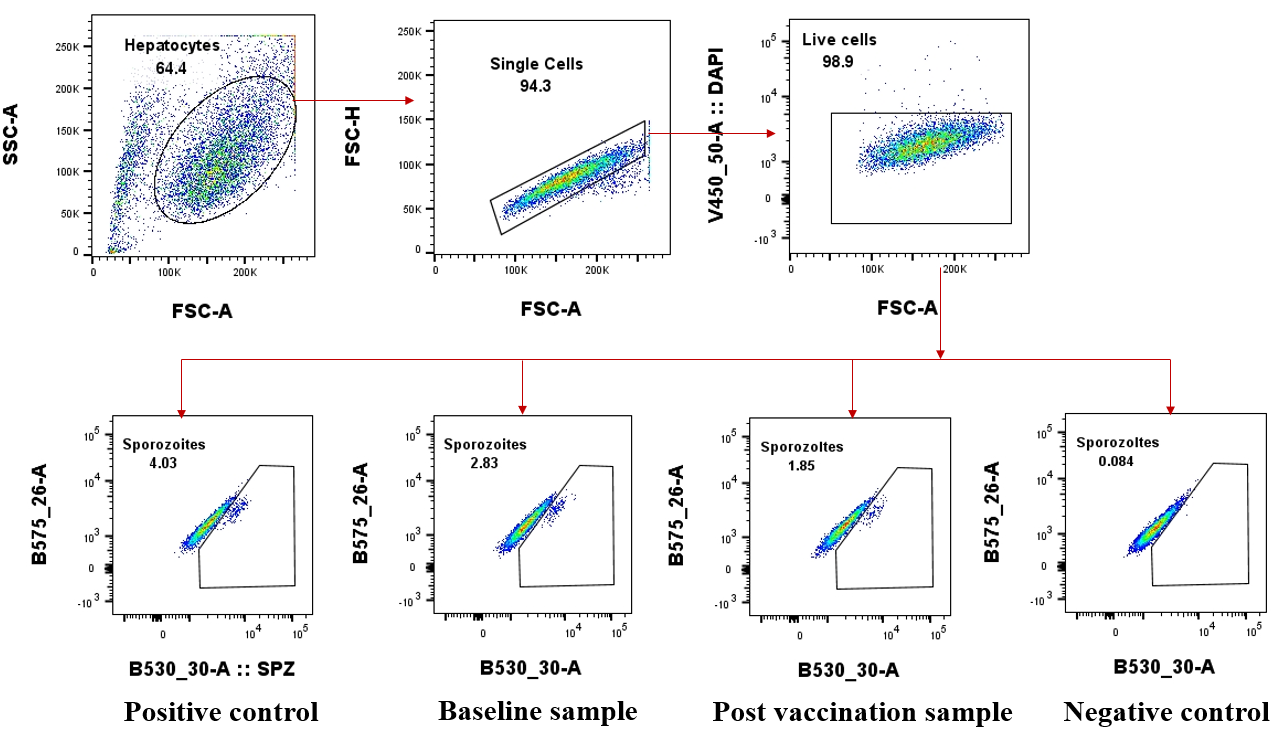


**Supplementary Figure 1: Gating strategy for detection of *P. berghei* infected hepatocytes cells.**

The hepatocytes were determined using the Side scatter area (SSC-A) and Forward scatter area (FSC-A). The single cells were gated for using FSC-H and FSC-A. Using the life-dead stain, the live cells were gated for DAPI negative. The negative control sample was used to guide the gating for sporozoite-infected hepatocytes.

**Supplementary Table 1: Sample assayed per timepoint**

|  | 1A/B | 2B | 3A/C | 3B/D | 3E |
| --- | --- | --- | --- | --- | --- |
| Anti-schizont ELISA | D0 n=18 | D0 n=16 | D0 n= 13 | D0 n=16 | D0 n=14 |
| MSD and Standardized ELISA | D0 n=18 D84 n=18, D816 n=15 | D0 n=16 D84 n=16, D816 n=16 | D0 n= 18, D84 n=18, D456 n=16 | D0 n=18, D84 n= 18, D456 n=18 | D0 n=15, D84 n= 15, D456 n=14 |
| C1q ELISA | D84 n=18 | D84 n= 16 | D84 n=18 | D84 n=18 | D84 n=15 |
| ISI | D0 n=13, D84 n= 13 | D0 n=3, D84 n=9 | D0 n=0, D84 n= 9 | D0 n=3, D84 n=9 | D0 n=5, D84 n=15 |
| Avidity ELISA -(R21 and NANP) | D84 n=18, D816 n=15 | D84 n=16, D816 n=16 | D84 n=17, D456 n=15 | D84 n= 18, D456 n=15 | D84 n= 15, D456 n=14 |
| NANP-IgA | D0 n=18 D84 n=18 | D0 n=2 D84 n=16 | D0 n= 13, D84 n=17 | D0 n=4, D84 n= 17, | D0 n=7, D84 n= 15 |
| NANP-IgM | D0 n=18 D84 n=18 | D0 n=4 D84 n=16 | D0 n= 5, D84 n=17 | D0 n=4, D84 n= 17, | D0 n=8, D84 n= 15 |
| R21-IgA | D0 n=18 D84 n=13 | D0 n=5 D84 n=16 | D0 n= 7, D84 n=17 | D0 n=7, D84 n= 18, | D0 n=14, D84 n= 15 |
| R21-IgM | D0 n=18 D84 n=16 | D0 n=5 D84 n=16 | D0 n= 5, D84 n=17 | D0 n=5, D84 n= 18, | D0 n=9, D84 n= 15 |

D0- Baseline, D84- one month post the third vaccination, D816/456-one month post booster dose. MSD-Meso-Scale Discovery (MSD) multiplex assay, C1q-complement-fixing (C1q), ISI-Inhibition of sporozoite invasion assay, ELISA-Enzyme-Linked Immunosorbent Assay. NANP-NANP6, (Asn-Ala-Asn-Pro) x 6. The number of samples assayed depended on the sample availability.

**Supplementary Table 2: Participant’s characteristics**

|  | **Group 1A/B** | **Group 2B** | **Group 3A/C** | **Group 3B/D** | **Group 3E** |
| --- | --- | --- | --- | --- | --- |
| **Category** | **Adults**  **(N=20)** | **Children**  **(N=17)** | **Infants**  **(N=18)** | **Infants**  **(N=18)** | **Infants**  **(N=15)** |
| Age in months (IQR) | 337.80 (309.67; 365.93) | 34.94 (28.53; 41.35) | 7.21 (6.31; 8.11) | 7.24 (6.28; 8.19) | 7.93 (6.90; 8.97) |
| Gender (%, male, n) | 75%, 15 | 47%, 8 | 47%, 8 | 35%, 6 | 60%, 9 |
| Vaccine dose (R21/MM) | 10µgR21/50µgMM | 10µgR21/50µgMM | 5µgR21/25µgMM | 10µgR21/50µgMM | 5μgR21/50μgMM |
| Anti-schizont IgG (Geo mean (95% CI)) | 11499 (7051 – 18752) | 738.1 (366.4 – 1481) | 41.55 (14.0 – 123.2) | 44.63 (19.1 – 104.3) | 34.08 (12.5 – 93.1) |

MM; Matrix M, Geo- Geometric mean NA: not applicable, IQR: Interquartile range, CI: Confidence interval

**Supplementary Figure 2: Anti-schizont IgG antibodies at baseline**

Anti-schizont IgG antibodies were measured by standardized ELISA at day 0 (baseline). The data shown is geometric mean ±95% CI. Kruskal-Wallis test with Dunn’s correction for multiple comparisons was used to determine the significance between the groups. Only p<0.05 was considered significant. The y-axis is in logarithm 10 scale.

**Supplementary figure 3: R21 antibody induced kinetics**

R21/Matrix-M vaccine induced antibodies a) Anti-R21, b) anti-NANP and c) anti-C-term total IgG geometric mean (±95% CI) as measured by ELISA. The Y axis shows the geometric mean (±95% CI) of antibody titres are specified as log10 ELISA unit. Horizontal dotted lines show the highest antibody levels attained, and the vertical dotted line indicates vaccination time points. X axis shows the time course by days. V1, vaccination 1, V2, vaccination 2, V3, vaccination 3. Booster dose was administered at 1 year for infants and at 2 years for adults and children. NANP-NANP6, (Asn-Ala-Asn-Pro) x 6, IgG, immunoglobulin G

**Supplementary Figure 4: Percentage durability rate of R21 vaccine-induced antibodies post-primary and booster dose.**

The percentage durability after primary vaccination was calculated by dividing the antibodies at 2 years (in children and adults group) or 1 year (in infant groups) by the peak antibodies (day 84), then multiplied by 100. The percentage durability after the booster dose was calculated by dividing antibodies 1-year post booster dose (i.e. 3rd year for the children and adults and 2nd year for the infants) divided by antibodies at one-month post booster dose (Day 456 for infants or Day 816 for adults and children), multiplied by 100). Kruskal-Wallis test with Dunn’s correction for multiple comparisons was used to compare more than two time points or groups, and determine the significance between the timepoints or groups. Comparisons between two time points or groups were conducted with Mann-Whitney tests. Only significance is indicated where p<0.05 The Y axis shows the median percentage (±95% CI) of antibody titres measured by ELISA (Supplementary Figure 3). Group 1A/B adults (10μg R21/50 μg Matrix-M), 2B children (10μg R21/50 μg Matrix-M), 3A/C infants (5μg R21/25 μg Matrix-M), 3B/D infants (10μg R21/50 μg Matrix-M), and 3E infants (5μg R21/50 μg Matrix-M).

**Supplementary Figure 5: Comparison of anti-R21 and anti-NANP IgA and IgM by age and by vaccination dose**

R21/MM vaccine-induced anti-R21 and anti-NANP IgA and IgM antibodies geometric mean (±95% CI) as measured by ELISA. The Y axis shows the geometric mean (±95% CI) of antibody titres are specified as log10 ELISA unit. Kruskal-Wallis test with Dunn’s correction for multiple comparisons was used to compare more than two time points or groups, and determine the significance between the timepoints or groups. Comparisons between two time points or groups were conducted with Mann-Whitney tests. Only significance is indicated where p<0.05. Sera was tested in triplicate.Group 1A/B adults (10μg R21/50 μg Matrix-M), 2B children (10μg R21/50 μg Matrix-M), 3A/C infants (5μg R21/25 μg Matrix-M), 3B/D infants (10μg R21/50 μg Matrix-M), and 3E infants (5μg R21/50 μg Matrix-M). NANP-NANP6, (Asn-Ala-Asn-Pro) x 6

**Supplementary Figure 6: Pre- and post-vaccination ISI correlations**

a) Pre- and post-vaccination percentage of blocked infection as measured by Inhibition of sporozoite assay (ISI). Comparison between Day 0 (baseline) and Day 84 (1-month post-vaccination) was computed by the Mann-Whitney tests. b-c) correlation between percentage blocked infection anti-C terminus and anti-R21 IgG antibodies. The correlation was computed with Spearman rank correlation. Group 1A/B-10μg R21/50μg Matrix M, group 2B- 10μg R21/50μg Matrix M, group 3A/C 5μg R21/25μg Matrix M, group 3B/D10μg R21/50μg Matrix M and group 3E 5μg R21/50μg Matrix M

**Supplementary Figure 7: R21-induced IgG antibody avidity and correlations.**

The Avidity index is reported as the measure the molar concentration of NaSCN needed to reduce the OD405 to 50% of that without NaSCN. a-c)Avidity was measured at D84 (one month post the third vaccination), D816 (one month post booster dose for adults and children) and D456 (one month post the booster dose in infants). a) adults-10μg R21/50μg Matrix M, b) children- 10μg R21/50μg Matrix M c) infants-5μg R21/50μg Matrix M, d) infants-10μg R21/50μg Matrix M and e) infants-5μg R21/50μg Matrix M. If samples at particular time point gave negative results for antigen-specific total IgG responses using ELISA, or if these responses were insufficient for analysis, the avidity was not included in the analysis. Individual responses are shown, plus the median and 95% CI. Kruskal-Wallis test with Dunn’s correction for multiple comparisons was used to compare more than two time points or groups, and determine the significance between the timepoints or groups. Comparisons between two time points or groups were conducted with Mann-Whitney tests. Only significance is indicated where p<0.05. d-e) correlation between the avidity index and the respective IgG antibodies at day 84. The correlation was computed with Spearman rank correlation. NaSCN; sodium thiocyanate, NANP-NANP6, (Asn-Ala-Asn-Pro) x 6
